# Epidemic Dysentery

**Published:** 1849-11

**Authors:** 


### EPIDEMIC DYSENTERY.

Dysnetery has prevailed in an epidemic form in many sections of the country, during the past summer, in some even exceeding the cholera in fatality. We are promised some papers on the subject by correspondents who witnessed the disease, and should be glad to receive communications respecting it from all who are able to contribute original observations as to its rise, pathology or treatment.

